# Street Viewer: An Autonomous Vision Based Traffic Tracking System

**DOI:** 10.3390/s16060813

**Published:** 2016-06-03

**Authors:** Andrea Bottino, Alessandro Garbo, Carmelo Loiacono, Stefano Quer

**Affiliations:** Dipartimento di Automatica ed Informatica, Politecnico di Torino, Torino 10129, Italy; andrea.bottino@polito.it (A.B.); alessandro.garbo@polito.it (A.G.); carmelo.loiacono@polito.it (C.L.)

**Keywords:** road traffic monitoring, vehicle tracking, vehicle counting, motion estimation, autonomous systems, flow network

## Abstract

The development of intelligent transportation systems requires the availability of both accurate traffic information in real time and a cost-effective solution. In this paper, we describe Street Viewer, a system capable of analyzing the traffic behavior in different scenarios from images taken with an off-the-shelf optical camera. Street Viewer operates in real time on embedded hardware architectures with limited computational resources. The system features a pipelined architecture that, on one side, allows one to exploit multi-threading intensively and, on the other side, allows one to improve the overall accuracy and robustness of the system, since each layer is aimed at refining for the following layers the information it receives as input. Another relevant feature of our approach is that it is self-adaptive. During an initial setup, the application runs in learning mode to build a model of the flow patterns in the observed area. Once the model is stable, the system switches to the on-line mode where the flow model is used to count vehicles traveling on each lane and to produce a traffic information summary. If changes in the flow model are detected, the system switches back autonomously to the learning mode. The accuracy and the robustness of the system are analyzed in the paper through experimental results obtained on several different scenarios and running the system for long periods of time.

## 1. Introduction

With increasing urbanization and vehicle availability, traffic systems in urban areas encounter many challenges, such as congestion, accidents and violence [1]. Institutions have allocated many resources to find solutions to those problems, such as building new infrastructure, optimizing traffic lights and rescheduling vehicle itineraries. Given the costs of such solutions, research on traffic flow monitoring systems, which aim to monitor and manage traffic streams, has also attracted much attention. Commonly-used sensors include loop detectors, magnetometers, radar sensors and microwave detectors.

In the meantime, more and more surveillance cameras have appeared in public areas. Compared to other sensors, video cameras have a lower cost, are less invasive and can produce richer information without effecting the integrity of the road [2]. However, as human operators are expensive and unreliable, optimal use of videos can be made only by automated surveillance systems, adopting efficient real-time computer vision algorithms [3,4].

Processing techniques of vision-based traffic flow monitoring are usually based on reliable and robust foreground vehicle detection. Services (such as traffic monitoring [5,6,7], anomaly or jam detection [8,9], traffic planning and forecasting [2]) are then based on tracking these foreground objects. Unfortunately, images obtained by most low-cost commercial camera systems are loaded with heavy noise, such as optical distortion and vibration. This noise is often difficult to eliminate. Moreover, the range of operational conditions (such as night, rainy, head light glare, dirt and occlusion) require robust techniques. Furthermore, most traditional approaches fail when a car accident or a temporary road construction modifies the original car stream.

### 1.1. Core Ideas

In this work, we propose a novel approach to produce a flow network and count the number of vehicles crossing a traffic area under video surveillance. As the application has to run on embedded systems with bounded computational resources, one of our targets is to minimize the computational effort, maintaining precision and reliability. To do that, we organized the application as a layered pipeline. The inner layer is the one receiving the input video stream. The outer layer is the one generating the final vehicle count or estimate. Each computational stage processes the input to provide more reliable pieces of information to the following phase, thus reducing the overall noise and the amount of data manipulated during each step of the process.

The application operates in two distinct working modes. Initially, it runs in *learning mode* targeting the construction of a proper *flow model*. In this stage, it first detects and tracks moving objects on a static background. Then, it identifies regions where notable movements have been detected. Finally, it divides them into lanes, *i.e.*, areas showing a coherent traffic flow, ready to be analyzed during the on-line operation mode. The training mode lasts until the traffic scheme is stable, *i.e.*, until all sources, streams (*i.e.*, directions and orientations) and sinks of the traffic scheme have been discovered. Once the flow model has reached convergence, the application automatically switches to the *on-line mode*. During this stage, the system uses the flow model to statistically analyze the traffic grid and to count (or estimate) the number of cars traveling in each single lane. The flow model is also useful to reduce the computational effort, as the system is able to focus its attention only on those sections of the image where lanes have been detected.

Furthermore, the graph-based model of the traffic patterns enables some high-level graph-based statistical evaluation, such as the one usually computed on standard flow networks (e.g., main and partial vehicle flows, capacity, bottlenecks, average speed, *etc*.).

As a final remark, notice that our two-phase system is able to switch back to the learning mode whenever a significant variation in the traffic patterns (due, for instance, to road works) has been detected.

### 1.2. Contributions

Our method shares with previous approaches several aspects and state-of-the-art methodologies. Nevertheless, it also presents several new characterizing features. Among them, we would like to highlight the following contributions:

- Our layered system architecture is suitable for embedded applications, with real-time requirements and finite computational resources, such as computational power and memory or energy availability.
- The dual-mode operation (learning and on-line) makes the system auto-adaptive, *i.e.*, the application does not require any sort of initial setup. The system switches from the learning to the on-line mode automatically, as soon as it has been able to build a stable traffic flow configuration.
- The system, working in multi-threaded mode, is able to automatically detect changes in the traffic flow, such as the ones due to construction works or accidents. In those cases, the system switches back to the learning mode without the necessity of human intervention.
- The final traffic model produced is a complete flow graph representation of reality, which enables different kinds of statistical analyses of the underlying flow network.
- The implemented system is reliable, that is it produces results with errors smaller than a few percentage during several long in-the-field counting experiments. Moreover, errors stay below 17% also during adverse weather conditions.

To sum up, our traffic application is particularly suitable for low-cost, low-computational power, real-time embedded applications in which traffic conditions change over time. The final model is much more informative and complete than standard techniques, enabling object counting [5,7,10,11].

### 1.3. Paper Outline

The paper is organized as follows. Section 2 describes a few close related works. Section 3 illustrates our methodology by: (1) detecting moving objects on a steady background (Section 3.1); (2) tracking blobs in subsequent frames (Section 3.2); (3) building a flow model (Section 3.3); and (4) performing the final counting (Section 3.4). Finally, it concentrates on how to compute some statistical measures on the data previously gathered (Section 3.5). Section 4 includes our experimental evidence on a few roads, cross-roads and roundabouts, for long period of time. Finally, Section 5 concludes the paper with a few summarizing remarks and some possible hints about future works.

## 2. Related Works and Comparisons

Computer vision in the context of traffic surveillance addresses problems such as automatic lane finding, vehicle or pedestrian detection, tracking, traffic flow measurements and the representation, understanding and prediction of human behavior.

In object detection, video acquisition cameras are usually stationary. Initial approaches in this field [2] involve spatial, temporal and spatio-temporal analysis of video sequences. The detection principle is essentially based on the fact that the objects to be searched for are in motion.

For example, Bas *et al.* [10] and Chen *et al.* [5] count vehicles by extracting object features and tracking those features by estimating their distance from the camera or measuring their minimal distance between two temporal images. Fernández-Caballero *et al.* [12] monitor traffic behavior on freeways and highways to get information on different traffic parameters and to automatically detect accidents.

Buch *et al.* [3] suggest that the majority of the counting systems focus on highways, as cameras required for this analysis need to be mounted on high poles and, therefore, are difficult to install. Moreover, most of the systems possibly perform classification to gather more detailed statistics.

Tian *et al.* [4] discuss the main challenges of video processing techniques in traffic monitoring. Among those, special scenarios, such as the ones with abnormal lighting conditions (cloudy and rainy weather), nighttime vehicle detection, shadow detection and removal and vehicle occlusion in dense traffic conditions, are particularly difficult to deal with.

More recently, practical applications, such as Autoscope and Monitorix, have been proved to reach a higher level of real-time performance. For example, Zhou *et al.* [13] build and simulate a traffic system model to understand the effects of changes in road configurations.

Poorani *et al.* [14] concentrate on highway monitoring based on vehicles crossing a registration line. Attention is paid to avoid re-counting, even if the actual count can be performed together with other analysis, such as vehicle length classification, speed control, and so on.

Yu *et al*. [15] count vehicles using as a main statistical parameter the difference of the gray value between the current frame and the background. The algorithm firstly generates the background. Then, it analyzes observation windows on road lanes. After that, it counts vehicles, based on the variation of the parameter selected. Finally, it updates the background based on notable changes detected in the scene. Unfortunately, the results are not conclusive, as the authors report a unique and short counting session where the system counts 54 vehicles on a three-lane straight highway. Moreover, the method is based on observation windows (virtual loops) manually placed on the observation lanes.

Yin *et al*. [16] present a reliable vision-based traffic flow detection approach. After prototyping the dynamic background of the traffic scene and extracting the foreground contours by image subtraction, the authors identify vehicles by comparing the binary vehicle contours’ location and the current frame. Vehicle counting is performed by a discriminative method used to classify vehicles into different lanes. The experimental result shows accuracy close to 100% on four lanes of two different roads, counting up to 200 vehicles per lane. Nevertheless, the approach cannot define the lanes adaptively.

Xia *et al*. [17] use the expectation-maximization algorithm to improve a Gaussian mixture model-based background subtraction. In addition, the authors adopt a restoration method to remove noise and fill holes to obtain a more accurate object extraction. The authors present five counting sessions with high accuracy, *i.e.*, precision values beyond 93% when counting up to about 300 vehicles. The method is based on detection windows (virtual loops) whose position is quite critical. Moreover, their application does not perform any training activity or model building, and it does not adapt itself to changing scenarios. Counting activity is presented on single or multiple lanes, where virtual loops are manually placed and all vehicles are moving in the same direction.

## 3. An Autonomous Follower System

Our autonomous follower system is organized as a pipeline, whose main input is the image sequence acquired from the optical system, and the final output is a flow network of the traffic scenario analyzed. Each computational layer is aimed at processing its input to provide more reliable pieces of information to the following layer, thus making the overall system more robust and accurate. In order to allow the real-time execution on embedded systems, we designed the system: (i) trying to find the right balance between the complexity of the algorithm and its computational load; and (ii) leveraging on a multi-threaded environment to parallelize as much as possible the different computational layers.

**Figure 1:**
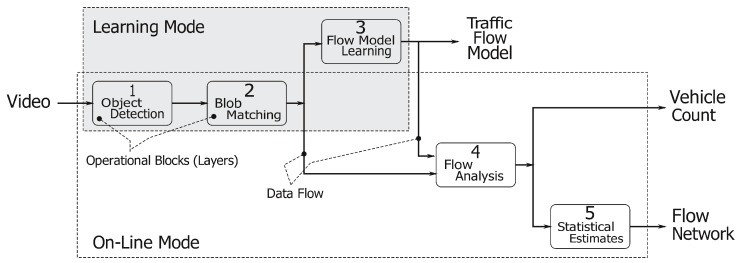
The pipeline structure of our follower system. The entire computation involves five different stages, namely object detection (Stage 1), blob matching (Stage 2), flow model learning (Stage 3), flow analysis (Stage 4) and statistical estimates (Stage 5).

The entire system pipeline is represented in Figure 1. The *object detection* stage (Layer 1) aims at segmenting the moving objects in the image stream. These objects are then tracked in the *blob matching* stage (Layer 2), and their motion is summarized into a compact representation named *track*. The *flow model learning* process (Layer 3) merges the available tracks to identify regions of the image where notable movements take place. Such regions are then organized in *lanes*, *i.e.*, areas of the image showing coherent traffic streams, which are the building blocks of the flow model. The *flow analysis* process (Layer 4) analyzes the output of the blob matching stage (Layer 2), taking into consideration the flow model computed by Layer 3, to count the number of cars traveling in each lane. Finally, the *statistical estimates* stage (Layer 5) gathers data coming from different lanes to build a flow network and to evaluate the overall traffic flow in the area under analysis.

**Figure 2:**
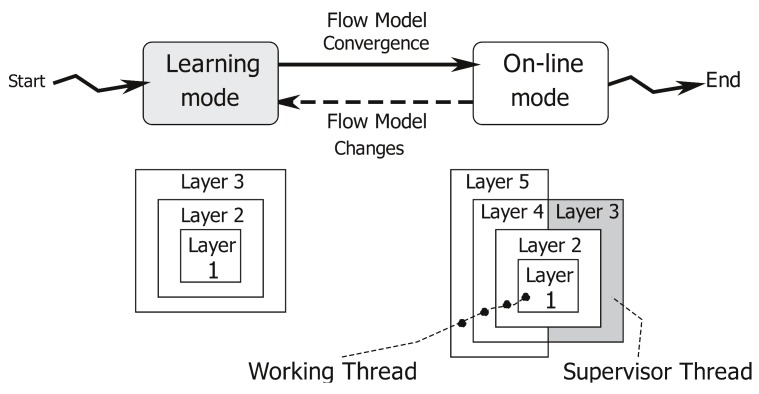
The operational view of our follower system: learning (gray box) and on-line (white box) stages. For each stage (*i.e.*, learning and on-line), the picture reports all computation layers (1. object detection; 2. blob matching; 3. flow model learning; 4. flow analysis; 5. statistical estimates) involved in the evaluation process.

As represented in Figure 1, the application has two different working modes: (1) The learning mode to create a traffic flow model; and (2) the on-line mode, which uses the traffic flow model to count the number of cars crossing the analyzed area and to produce a flow network. This operational view of our system is represented in Figure 2. The application starts in learning mode, running Layers 1, 2 and 3, and it creates a traffic flow model. When the traffic flow model has become stable (notice that a more precise definition of our concept of “flow model stability” will be given in Section 3.3.1), the system automatically moves to the on-line mode. In the on-line mode, Layers 1, 2, 4 and 5 are involved as the main working threads, whereas Layer 3 is run as a lightweight supervisor parallel thread. The working threads deliver the desired vehicles’ counting and produce a flow network useful for more detailed statistics. When the supervisor worker detects any traffic flow model modification, the application switches back to the learning mode to update the flow model itself before re-starting a new on-line counting phase.

**Figure 3:**
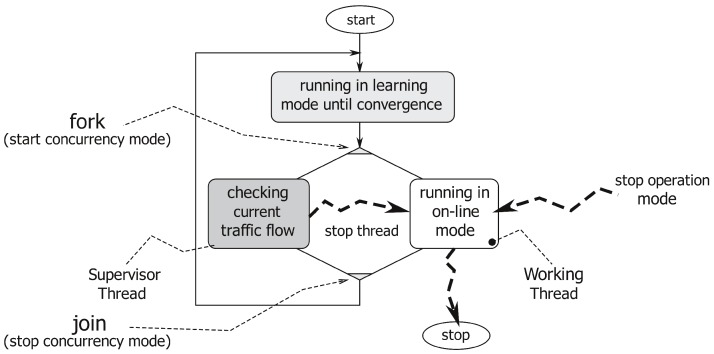
The concurrent logic of our system: moving from learning (gray box) to on-line mode (white box) stimulates a shift from the sequential to the concurrent mode. The darkest box run in the concurrent mode represents the lightweight supervisor thread.

Figure 3 describes this concurrency (multi-threaded or multi-worker) nature of our application. When the system switches from the learning mode to the on-line one, it activates a supervisor thread, which analyses possible modifications of the traffic flow model (such as the ones due to construction works). In brief, the supervisor works as follows. First of all, it identifies the lanes to which each track belongs (as described in Section 3.4.1). Then, if this set of lanes is not coherent with the flow model, it stores the discrepancies into a history image, a quantized version of the image space whose intensity is a function of recency and the duration of discrepancies. By thresholding this discrepancy map, the application identifies persistent variations of the flow model. These variations can trigger a switch from the on-line back to the learning mode.

The following sub-sections detail the different layers of our processing pipeline.

### 3.1. Layer 1: Object Detection

The first block of the pipeline manages the lower-level processing tasks, *i.e.*, the identification of the moving objects in the image stream. These objects are a super-set of those of interest for the system, since they might include both vehicles (cars, trucks, buses) and non-vehicles (people, animals, moving objects).

Since we are interested in detecting objects moving on a stationary background, segmentation has been based on the background subtraction approach proposed in [18]. This state of the art method relies on an adaptive background model whose pixels are described by a mixture of Gaussian distributions. Both the parameters and the number of components of the mixture are computed per-pixel on a training sequence of size *N*. Allowing each pixel to be associated with a different model, the algorithm improves its capability to carefully describe the local scene characteristics, and consequently, it provides an improvement to the overall robustness.

After the background model has been built, an image pixel is marked as foreground if it is sufficiently different from the corresponding pixel distributions in the model. The algorithm automatically adapts itself to slow changes in the background by updating the Gaussian mixture models according to the background pixels detected in the previous *N* frames. The parameter *N*, which is the same used to build the model, controls the adaptation rate of the algorithm.

**Figure 4:**
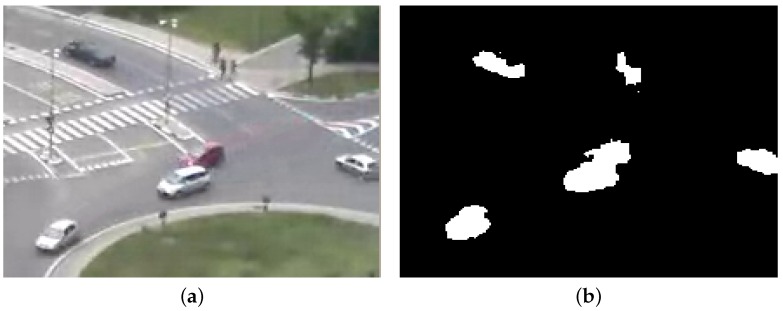
Object detection phase: (**a**) initial image; and (**b**) detected objects. Notice the top-right segmented objects, corresponding to a pedestrian group, which will be filtered-out in the following layer.

After segmentation, objects are extracted by first applying morphological operators (dilation and erosion) to the foreground mask and then identifying the connected components. An example is reported in Figure 4 on a roundabout scenario, which will be used as the running example throughout the entire paper.

### 3.2. Layer 2: Object Tracking

In this module, moving objects are tracked along the video sequence, and their temporal motion patterns are summarized into *tracks*. Each detected object can be assigned to an available track or can generate a new track. Conversely, tracks with unassigned objects are closed (*i.e.*, the corresponding tracked object has been lost).

The parameter setting and the algorithm flow vary according to the system state. In learning mode, we must reduce as much as possible the amount of noisy or ambiguous data that are fed to the flow model learning layer, and thus, we impose stricter matching constraints. On the contrary, in on-line mode, we relax these constraints, since the availability of an accurate flow model helps the following layers to better cope with segmentation problems without removing pieces of information that are potentially relevant for the counting task.

Regardless of the system state, each track describes an object trajectory with the following data:

- the temporal sequence of the object positions;
- the actual bounding box of the tracked object;
- its state (active or closed);
- its *robustness*, a parameter between zero and 100 expressing the reliability of the track.

Summarizing, our approach tries to match blobs (*i.e.*, objects detected in the current frame by Layer 1) with tracks (*i.e.*, objects tracked in the previous frames) by exploiting spatial proximity and motion congruence. More in detail, segmented objects are assigned to a track if their bounding boxes intersect the track bounding box. As a consequence, if an object has been assigned to a single track, (i) the centroid of the assigned object is stored into the track motion history; (ii) the track robustness is increased by a factor $\delta_{ass}$ using saturation arithmetic ($\delta_{ass}=5$ in learning mode and $\delta_{ass}=10$ in on-line mode); and (iii) the track bounding box size is set to the running average of those of the last $w_{bbox}=10$ assigned objects. Unassigned tracks are marked as closed. As for unassigned objects, each of them contributes to the creation of a novel track, whose initial robustness is equal to zero.

Then, according to the system state, we treat in different ways the cases of (i) multiple objects assigned to the same track and (ii) groups of merging tracks, *i.e.*, sets of tracks whose updated bounding boxes reciprocally intersect.

In learning mode, since these cases are a possible source of ambiguities, we simply discard multiple assignments and mark the merging tracks as closed.

In on-line mode, we preserve both pieces of information. This is done, for multiple objects, by averaging their position and bounding box in order to obtain a unique sample to be assigned to the track. In case merging tracks have been identified, we maintain as open only the track with the greatest robustness, marking as closed all of the other ones. The centroid and bounding box of the surviving track are set to the average of those of all of the merging tracks.

**Figure 5:**
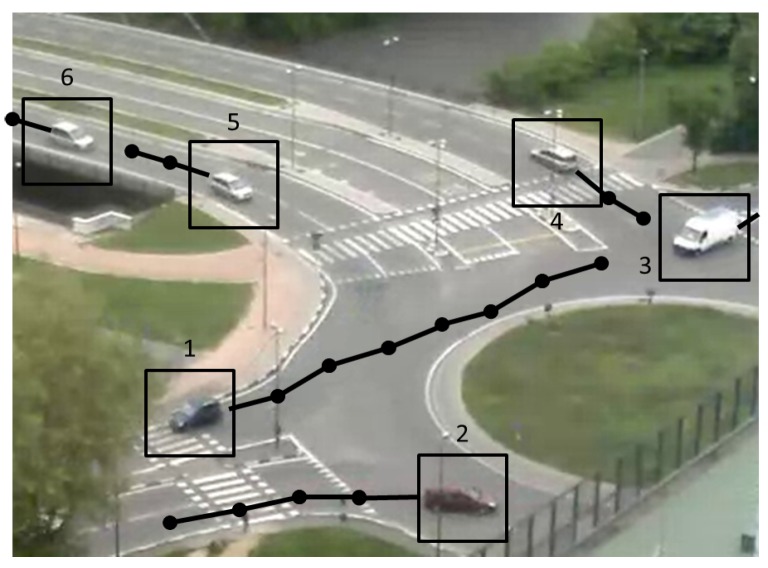
Objects tracked along a traffic sequence. The main dots on the curves highlight trajectory points used by the tool to evaluate vehicle routes.

The output of the algorithm is the list of closed tracks in the current frame. Figure 5 shows all detected tracks overlaid to a video image of the roundabout.

Clearly, the performances of this second layer are affected by those of the object detection module. Besides abrupt weather changes, which require time to be incorporated into the background model and thus produce large segmentation errors, segmentation results can include noise, non-vehicle objects and occlusions. Since we noticed that small segmentation errors and non-vehicle objects are characterized by a smaller area with respect to actual vehicles, we found that a simple threshold on this parameter was sufficient to prune them. As for vehicle occlusions, which cause two objects to be represented as a single one, we noticed that occlusions are usually resolved in short time intervals (after which the two vehicles are again identified as separate objects). Thus, we deemed it preferable to delegate occlusion management to the following processing layers, which can exploit for this task pieces of information at higher levels of abstraction.

### 3.3. Layer 3: Flow Model Learning

The purpose of the flow model learning layer is two-fold: first, identify the *lanes*, *i.e.*, image regions showing coherent traffic streams, and then provide a compact representation for them, which will be exploited by the flow analysis layer to reduce counting errors. We stress the fact that while in other approaches (such as [19,20]), the set of lanes to be monitored is defined interactively by the operators of the traffic management center, in our work, we provide an automatic algorithm for their identification and manipulation. The outline of the flow model learning phase is the following:

1. The image is first divided into sub-regions (*cells*), and then, the motion vectors that best summarize the traffic patterns in each cell are computed exploiting the track data obtained from the previous layer.
2. When cell data become stable, *i.e.*, when the analysis on new tracks does not provide relevant changes of their values, lanes are identified by clustering cells according to their flow information.
3. A compact representation of each lane is computed.

These steps are detailed in the following.

#### 3.3.1. Motion Vector Computation

First of all, we divide the image into a uniform grid of (w,h) cells, where *w* and *h* are set according to the frame size in order to find the proper balance between the rate of convergence of the model and its resolution. Each cell contains a set of *directions* summarizing the traffic flows through the cell. The possibility to consider different flow directions in a cell allows a proper modeling of cells located in road junctions. Each cell direction is characterized by its orientation versor, the list of samples used to compute it and the most recent time code of these samples. All cells are initialized with an empty direction set.

Grid data are updated according to the list of closed tracks identified at the previous layer. In order to reduce noise in the final flow model, we only process tracks having high robustness (for example, above 70 for at least 50% of their existence). Then, to smooth the noise in the track data, we approximate each track with a piece-wise linear curve whose vertices are the first, the one in the *k*-th position and the last one over time of the track points. The grid cells intersected by this approximating poly-line are then found with a modified Bresenham algorithm [21], and for each of these cells:

- We estimate the local track orientation as (i) the versor of the poly-line segment intercepting the cell if the cell does not contain a poly-line vertex and (ii) as the average of the versors of the edges sharing the poly-line vertices included in the cell, in all other cases.
- The set of cell directions is updated with the computed local track orientation as follows:
  - If the set is empty or the cell does not contain any direction similar to the sample, the sample is added to the set as a novel direction.
  - If the cell contains a direction similar to the sample, the sample is assigned to this direction.
  In the first case, the condition for the sample and a cell direction to be similar is that the angle between them is lower than a threshold $t_{\alpha }$. We experimented with values $t_{\alpha }\in \left[10^{\circ },30^{\circ }\right]$. In the second case, the direction versor is computed as the running average of the last *k* assigned samples (such as k∈[10,100]).
- After updating the cell directions, if two directions in a cell are found similar, according again to $t_{\alpha }$, they are merged in a unique direction. Furthermore, if a cell has not been “refreshed” for a long period (usually, several minutes), it is removed from the model, being considered as an outlier.

**Figure 6:**
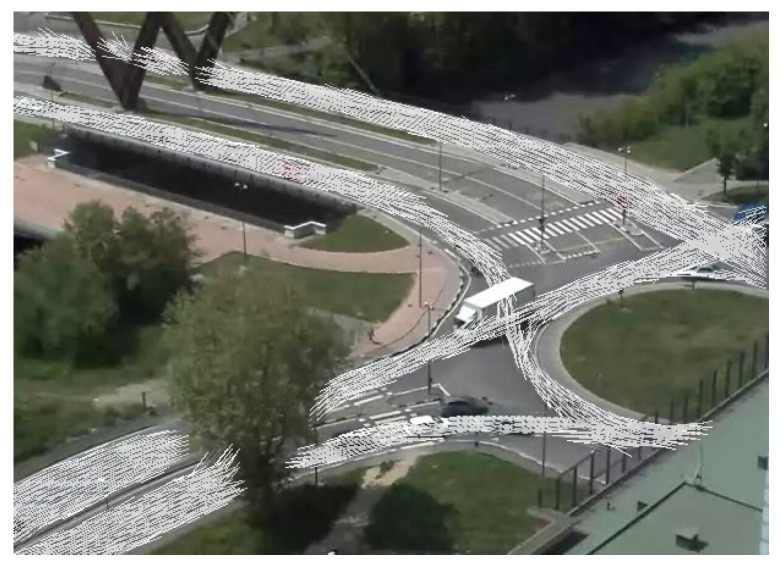
Learning the flow model: the directions stored in the grid cells are super-imposed on a camera image.

This process is iterated until the model becomes stable, *i.e.*, until no relevant changes in the cell motion vectors have been detected. More in detail, we assign to each change in the cell directions a weight, which is higher when the change is related to the introduction of a novel direction and lower when it simply contributes to a direction update. When the sum of these contributions over time become smaller than a threshold, the cell is considered stable. When the overall number of stable cells reaches a specific percentage, the entire flow model is considered stable, and the application switches to the on-line mode. Our experimental analysis shows that the model usually converges after from a few hundreds to a few thousands of vehicle tracks have been detected. This usually requires from tens to hundreds of minutes depending on weather and traffic conditions and their variations over time. To be as fast and accurate as possible, the learning phase should be performed in standard weather conditions and standard traffic density. Very low traffic or poor illumination or weather conditions can affect the duration of this phase or the quality of the final model. An example of a temporary flow model built by the system is shown in Figure 6.

Notice that the learning phase does not have to be repeated if there are no abrupt traffic changes, if the position of the camera does not change or there are illumination or weather changes. As a final remark, recall that a lightweight version of this process is also executed during the on-line operation mode. In that case, the goal is to let the supervisor thread properly react to variations in the traffic scheme, as introduced at the beginning of Section 3.

#### 3.3.2. Lanes’ Detection and Representation

Lanes are identified by clustering neighboring cells whose motion vectors share similar directions. Clustering works as follows.

- We scan the cell grid looking for the first cell that has not been yet assigned to any lane, and for each direction stored in this cell, we create a new cluster seed.
- Then, we apply a region growing algorithm where the condition for adding cells to the region is again based on the similarity between the directions of neighboring cells. In this case, a stricter threshold $t_{\beta }<t_{\alpha }$ is used. Cells having more than one motion vector might belong to different lanes.
- Finally, treating the cluster cells as pixels, we apply morphological operators, and we look for the lane source and sink. This is done by viewing the lane as a vector field and computing an approximation of its divergence in each cell. The cell with the maximal divergence is picked as the source, the one with the minimal divergence as the sink.

**Figure 7:**
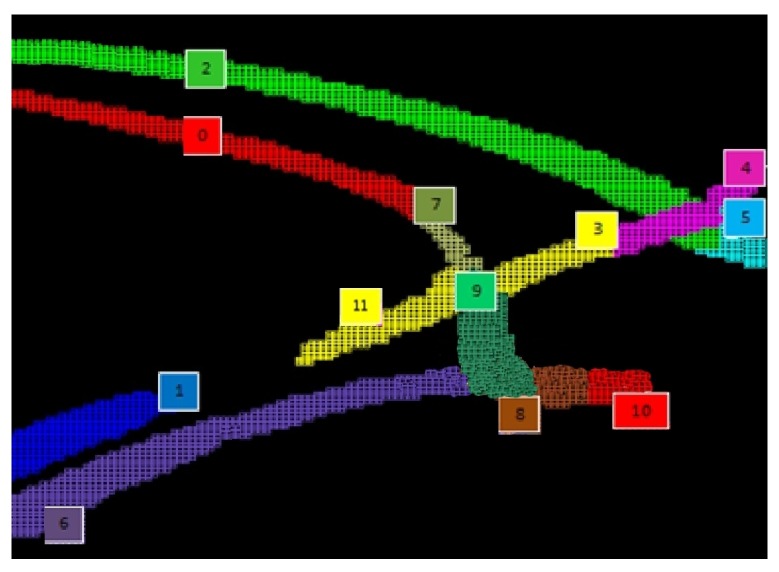
Detecting and representing lanes on the roundabout running example.

It should be highlighted that lanes in the flow model are not necessarily coincident with physical lanes, since the algorithm is affected by road occlusions in the images due to obstacles, like trees or houses. In this layer, after all lanes have been identified, we try to remove small occlusions, such as the ones affecting the down-most lanes of Figure 6. This is done defining merging rules based on both geometric proximity and flow continuity. Applying such rules in the previous example, it is possible to obtain a single lane labeled as Lane 6 in Figure 7. Larger occlusions will be analyzed by Layer 5.

#### 3.3.3. Representing Lanes in a Compact Way

As the final step, each lane is transformed into a compact representation that will be effective at improving the accuracy of the vehicle counting process. This compact representation is a piece-wise linear curve *L*, whose vertices are identified in the following way. The first point of *L* is the lane source, and its direction is taken as the initial reference direction. Then, we visit the source neighboring cells in a breadth-first order. When we found a lane cell whose direction makes an angle greater than a certain value (usually small) with the reference direction, we store this point in a list, and we update the reference. When all cells have been visited, we append to the list the position of the sink. The curve *L* is then parametrized by its arc length as L(s). Therefore, L(0) represents the image position of the lane source and L(1) that of the lane sink.

Concluding, the final flow model is represented as a list of lanes, each characterized by a list of cells and a curve L(s), which will be the target of our subsequent counting activity.

### 3.4. Layer 4: Flow Analysis

This layer is in charge of counting the number of cars traveling in each lane, and we recall that it is active only during the on-line operational mode. The inputs of this module are the flow model (*i.e.*, the list of identified lanes) and the per-frame list of closed tracks obtained by the object tracking module (Layer 2), and the output will be the number of vehicles traversing the lane.

We underline the fact that tracks are affected by possible segmentation and tracking errors. For instance, since we did not consider any filtering in the tracking process (*i.e.*, Kalman or particle filtering) to reduce the computational burden, if the tracked object is missed in some frames or two vehicles occlude each other in a certain temporal window, the same moving object might be represented by different tracks or a track segment might correspond to more than one vehicle. This is a relevant issue, since it affects the continuing accuracy, and it has been managed in our approach as follows.

#### 3.4.1. Introducing the Counting Trajectories

First, as the initial pre-processing step, since each track can belong to different lanes, we split the track into sub-tracks according to the lane labels of the cells intersected by the tracks. If a cell contains multiple lane labels, we pick the one of the lane having a direction closer to the track one.

The key factor that allows our approach to moderate the effects of segmentation and tracking problems is that we process the vehicle motion into a 2D space, representing the distance traveled along the lane during time, rather than in the original 3D (image position, time) reference system.

To do this, we convert the 3D sub-tracks defined in the (x,y,t) space of temporal image positions into *counting trajectories* defined in a 2D space. Such counting trajectories are obtained transforming each sub-track point $P_{i}\left(x_{i},y_{i},t_{i}\right)$ into a point $P_{i}^{\prime }\left(t_{i},s_{i}\right)$ by first projecting $P_{i}$ on the curve L(s) of its reference lane and then computing the value *s*, with s∈[0,1], at the projection point. The *counting trajectory* T(t) is then defined as the quadratic curve approximating the set of points $P^{\prime }\left(t,s\right)$, which are called the *support* of T(t). Clearly, any counting trajectory is monotonically increasing. The rationale of choosing a quadratic approximation is that we found it more suitable than a linear approximation to deal with vehicle acceleration and image perspective effects affecting the track data.

We then define a counting trajectory as *complete* if it has a support starting at s=0 and ending at s=1, otherwise the trajectory is defined as *incomplete*. We underline that, actually, since the transformation of tracks into counting trajectories can suffer from approximation errors, we relax the completeness constraints as, respectively, s<ϵ and s>1-ϵ; the value of *ϵ* is automatically adapted to the lane characteristics.

#### 3.4.2. Processing the Counting Trajectories

In the following, for the sake of brevity, we will simply refer to the counting trajectories as trajectories. How can these trajectories be used to improve the counting accuracy? We recall that we can have two different problems: (i) a vehicle crossing the lane that is represented by different incomplete trajectories; and (ii) a trajectory or a trajectory segment representing the motion of multiple vehicles.

In order to deal with the first problem, we can try to find incomplete trajectories that possibly describe the motion of a unique object. Two incomplete trajectories $T_{1}$ and $T_{2}$ are merged if $T_{1}$ correctly approximates the support of $T_{2}$, and vice versa. We have that $T_{i}$ correctly approximates $T_{j}$, if the maximal distance between every point $P^{\prime }$ of the support of $T_{i}$ and the curve $T_{j}$ is lower than a pre-defined threshold. The approximating curve for the merged trajectory is then recomputed using as support all of the points of the supports of $T_{1}$ and $T_{2}$. This process is repeated until no more incomplete trajectories can be merged.

**Figure 8:**
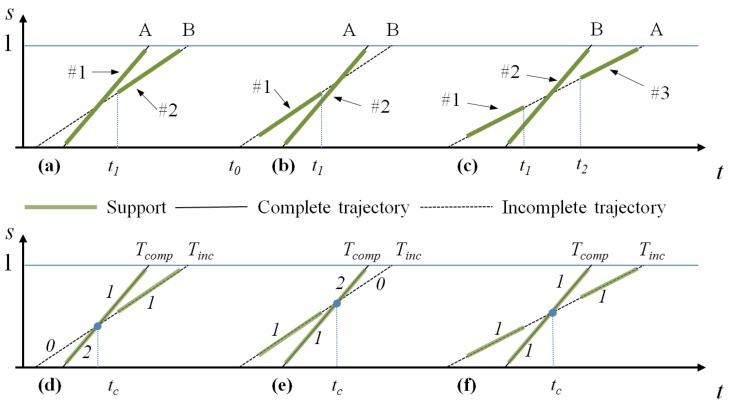
Counting trajectories’ processing. First row (**a**–**c**): three different scenarios involving the intersecting trajectories of two vehicles, A and B; second row (**d**–**f**): for each of the previous scenario, we show the complete (continuous lines) and incomplete (dashed lines) trajectories, the trajectory support (thicker segments) and the vehicle counters for the final trajectory segments.

A second improvement of the counting accuracy consists of trying to estimate the real number of vehicles represented by a complete or incomplete trajectory. To introduce this step, imagine the three scenarios depicted in Figure 8a–c.

1. Scenario 1 (Figure 8a): Vehicle A enters the lane at time $t_{0}$, partially hiding Vehicle B that proceeds at a similar speed until A definitively overtakes B at time $t_{1}$, and then, the two vehicles exit the lane at different times. In terms of tracks, we have a first Track #1 that describes the motion of both A and B until $t_{1}$ and of Vehicle A only for $t>t_{1}$, and a second Track #2 starting at $t_{1}$ describing the motion of Vehicle B. In terms of trajectories, Track #1 originates a complete trajectory and Track #2 an incomplete trajectory.
2. Scenario 2 (Figure 8b): Two vehicles, A and B, enter the lane at different times, and from time $t_{1}$, they proceed side by side until the lane ends, with Vehicle A partially hiding Vehicle B in the images. This scenario results in a first complete Trajectory #1 representing Vehicle A for $t<t_{1}$ and both vehicles for $t>t_{1}$ and a second incomplete Trajectory #2 ending at $t_{1}$ and representing only B.
3. Scenario 3 (Figure 8c): Vehicle A enters the lane at time $t_{0}$ with a speed $v_{A}$; B enters the lane at $t>t_{0}$ with a speed $v_{B}>v_{A}$. B overtakes A in the temporal interval $\left[t_{1},t_{2}\right]$, where it also hides A in the camera images. As a result, we obtain three trajectories, #1, incomplete, in $\left[t_{0},t_{1}\right]$ describing the motion of A, #2, complete, describing the motion of B for $t<t_{1}$ and $t>t_{2}$ and of A and B in $\left[t_{1},t_{2}\right]$, #3, incomplete, $t>t_{2}$, describing again the motion of A. Then, the analysis of Trajectories #1 and #3 finds that they can be merged, reducing the final number of trajectories to two.

In all of these scenarios, it is clear that the final number of trajectories obtained is equal to the number of vehicles traversing the lane. Equivalently, we can assume that each trajectory corresponds to a vehicle. If this statement were true, for each trajectory *T*, we could store the values $t_{i}$ and $t_{o}$, *i.e.*, the estimated time the vehicle enters ($T(t_{i})=0$) and exits ($T(t_{o})=1$), the lane to compute statistics on the vehicles driving through the lane.

However, such statistics could be affected by small errors when computed at a specific time *t* since: (i) a trajectory, or a trajectory segment, can indeed describe the motion of more than one vehicle; and (ii) if the trajectory is incomplete, its $t_{i}$ and $t_{o}$ are only estimates of the real enter and exit time of the vehicle.

To overcome this problem, we first identify possible trajectory segments, and then, we assign to each segment a vehicle counter initialized to one. Such segments are determined by the intersection between trajectories (clearly, since complete trajectories cannot intersect each other, we must consider only the intersections between complete and incomplete trajectories and those between incomplete trajectories).

In the first case, a complete trajectory $T_{comp}$ intersects an incomplete trajectory $T_{inc}$ at $t_{c}$. At the intersection point, both trajectories are split into two segments, the left segment $\left[t|t<t_{c}\right]$ and the right segment $\left[t|t\geq t_{c}\right]$. Only three cases are possible, namely those corresponding locally to the three scenarios previously introduced:

- Scenario 1 (Figure 8d): All of the support of $T_{inc}$ lies in the right segment ($t\geq t_{c}$); then, the counter of the left segment of $T_{inc}$ is decremented by one, and the counter of the left segment of $T_{comp}$ is incremented by one. That is, two cars are detected and tracked as a single object until $t_{c}$ and represented by a trajectory segment with Counter 2 ending at $t_{c}$; after $t_{c}$, the two vehicles are tracked as separate objects, resulting in two trajectory segments having Counter 1.
- Scenario 2 (Figure 8e): All of the support of $T_{inc}$ lies in the left segment ($t<t_{c}$); then, the counter of the right segment of $T_{inc}$ is decremented and the counter of the right segment of $T_{comp}$ is incremented. That is, two vehicles are counted as separate objects until $t_{c}$ and then as a unique object after $t_{c}$.
- Scenario 3 (Figure 8f): The support of $T_{inc}$ lies in both the right and left segment of $T_{inc}$ (*i.e.*, $T_{inc}$ has been obtained merging two incomplete tracks). This situation happens when two vehicles are partially tracked as a single object during the path trough the lane, but they enter as separate objects and exit as separate objects. Thus, no segment counter needs to be updated.

Then, any trajectory segment having a null counter is discarded, and the whole process is repeated, until no more intersections between trajectories can be found.

For the sake of brevity, we do not detail the cases related to the intersection between incomplete trajectories, which easily follow from the ones we just described.

Concluding, after trajectories have been created and processed, we are able to tell at each moment in time *t* the exact number of vehicles actually traversing a lane and the total amount of vehicles that entered and exited the lane.

It is clear, however, that consolidated values can be available only after all of the tracks that possibly influence each other have been collected. Since, according to the lane, a varying number of frames are necessary for a vehicle to cover its entire trajectory, we consider solid the statistics at time *t* if they have been computed at time t+Δt, with Δt heuristically chosen to be fit to all scenarios.

### 3.5. Layer 5: Statistical Analysis

So far, we have defined the lanes as the elements where the traffic flow can be computed, and we have provided a robust method for estimating such flow. However, each lane is still considered individually, and a structure capable of describing the relations between lanes and providing summary results computed over all of the observed area is missing.

The aim of the statistical analysis layer is to build such a structure by creating a flow network capable of correlating data coming from the individual lanes. In graph theory, a flow network is a directed graph where each edge receives a flow. As for nodes connecting edges, the amount of flow into a node equals the amount of flow out of it, unless it is a *source*, which has only outgoing flow, or a *sink*, which has only incoming flow.

In our case, the lanes are the edges of the flow network, and the nodes, *i.e.*, the connections between lanes, can be obtained from the geometric properties of the lanes. However, due to occlusions, there can still be real lanes represented by two or more logical lanes, physically too distant to be geometrically connected by Layer 3. As an example, consider Lanes 1 and 11 in the bottom left corner of Figure 7. To overcome this issue, Layer 5 tries to argue logical lane proximity and to reconstruct the missing links exploiting both the input and output flows of the logical lanes and their relative position.

**Figure 9:**
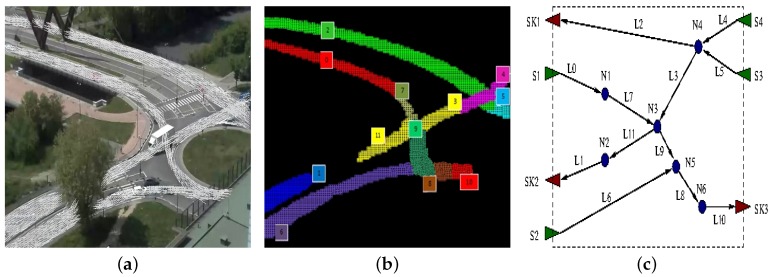
Building a flow-network on the (partially occluded and interrupted) lanes on the roundabout running example. The flow model of Figure 6 is reported in (**a**) for convenience; the lanes structure is reported in (**b**); in the flow network of (**c**), green triangles represent sources (Si), red ones sinks (SKi), and circles are used to represent generic connection points (Ni). Lanes are represented as black edges.

A rough idea of how this process works when applied to the scenario of Figure 7 is depicted in Figure 9, which shows the flow model obtained from Layer 3 (Figure 9a, same as Figure 6), its logical lanes (Figure 9b, same as Figure 7) and the final flow network (Figure 9c). In Figure 9c, triangles represent flow network sources (green triangles) and sinks (red triangles), whereas circles stand for nodes. Lanes are represented as edges, which are numbered using the same naming scheme of lanes and eventually annotated with the actual car flow direction. It is easy to notice that the lanes L11 and L1 can be connected introducing node N2 since the outgoing flow of the lane L11 is quite similar to the incoming flow of the lane L1, thus overcoming the occlusion due to the tree (see Figure 9a). Similarly, the lanes L4, L5, L2 and L3 have a net balance close to zero, and they result in being incoming (L4 and L5) or outgoing edges (L2 and L3) of node N4.

**Figure 10:**
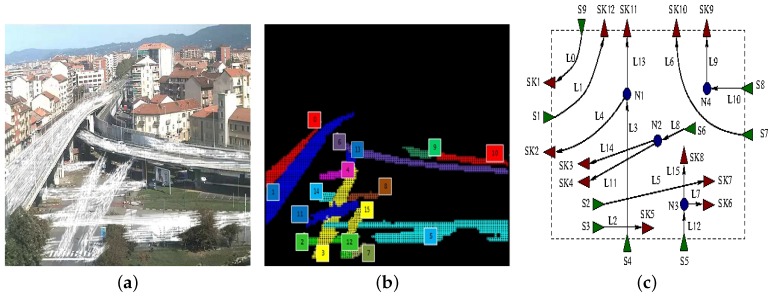
Building a flow-network for the cross-road running example: Flow model (**a**); lanes (**b**); and flow network (**c**).

Another example is the one in which the traffic scheme is more complex or partially undefined due to the fact that a portion of the overall picture remains uncovered. In this case, after the initial training section, Layer 5 tries to connect dangling sources and sinks found inside the picture. An example of such a behavior is illustrated in Figure 10. While the presence of node N4 is guessed following the same reasoning previously described (as L9 and L10 are actually two segments of the same physical lane, which is partially occluded), the presence of node N1–N3 is guessed computing the overall net balance between source and sink flows in the same area.

**Figure 11:**
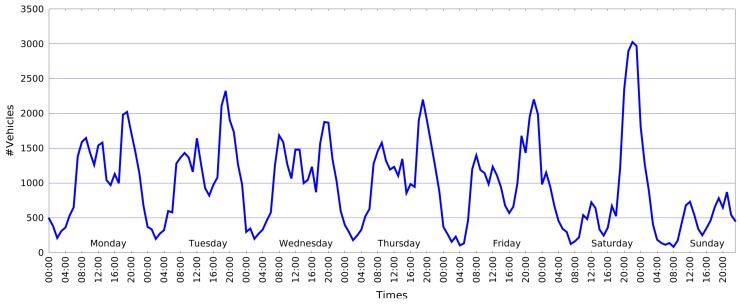
Roundabout weekly statistics.

Once the flow network has been computed, it is possible to obtain several statistics on single regions or on the entire area observed. As an example, in Figure 11, we report the number of vehicles crossing the roundabout scenario over an entire week. The graph shows that within the working days (from Monday to Friday), the number of vehicles is quite high during two hour ranges, *i.e.*, 07:00–09:00 a.m., 11:00 a.m.–01:00 p.m., and really high during the late evening, *i.e.*, 06:00–08:00 p.m. On the contrary, on Saturday evening, the plots present a peak at 10:00 p.m., and on Sunday, the number of vehicles is significantly lower.

## 4. Experimental Results

The system described in the previous sections has been successfully implemented and deployed, for over two years, on several locations in the area of Turin, a city in the northwest region of Italy.

The hardware configuration used for all tests includes the following devices:

- A CuBox-i4Pro embedded system [22] running Android; the CuBox-i is a compact micro-computer (a cube of $2^{\prime \prime }$ size) with a quad-core processor, a RAM of 2 GB, a GC2000 GPU and an external memory (on micro-SD) of 4 GB.
- A video-camera of the AXIS P13 Network Camera Series [23], having a varying resolution ranging from SVGA up to 5 Mpixel, including HDTV 720 and 1080 pixel video. The maximum frame rate is equal to 30 fps. The camera also provides features like wide dynamic range and day and night functionality, delivering good image quality in both conditions.
- A unique (centralized) quad-core workstation, for all statistical analysis performed at Layer 5, with a CPU frequency of 3.4 GHz, and equipped with 8 GB of main memory.

In the following sub-sections, we will present a detailed analysis discussing:

- The performances of our application running on the embedded CuBox-i4Pro system compared to the ones gathered on the quad-core workstation (Section 4.1).
- The accuracy of the system in optimal operational conditions (Section 4.2).
- How different operational conditions (such as sunny, rainy or foggy weather) affect system accuracy (Section 4.3).
- The sensitivity of the processing pipeline to the parameters described in Section 3 (Section 4.4).
- Overall results and their accuracy, gathered on different scenarios for long periods (Section 4.5).

### 4.1. System Performance Evaluation

To a great extent, one of the targets of our application is to be able to manage complex scenarios, such as the one in which the system includes a network of video-cameras analyzing a close area. In this kind of application (as in on-line run-time applications), to minimize the amount of data sent over the network and to reduce the workstation workload, the portable terminal device should extract and deliver only those data that are essential to the problem at hand. For that reason, our application has been designed to run on embedded platforms during all main (on-line) computations (*i.e.*, all computations performed by Layers 1, 2, 3 and 4). In this way, only “compressed” information is transferred to the workstation. Moreover, the workstation is free to finalize the computation itself merging data coming from several cameras. In other words, in our scenario, only Layer 5 is designed to explicitly run on the workstation.

Anyway, for the sake of completeness, in this section, we briefly compare the performances of our application running on the CuBox-i4Pro embedded system (with limited resources) and the quad-core (much more powerful) workstation. Moreover, we here consider the learning phase already performed (and the traffic flow model available), and we concentrate on the result gathered in the on-line mode.

Table 1 reports the running time required by the different computation layers on the two selected platforms. We collect results by running the counting process for one hour overall, assembling back-to-back several videos with different traffic and weather conditions. For each run, we collect the CPU time, and we present its average value per video frame. Column Operation Layer indicates the computation layer of which we evaluate the time. Layers are inserted incrementally on successive rows of the table. Columns Workstation and Embedded reports average times (in milliseconds) for the quad-core workstation and CuBox-i4Pro, respectively. Notice that we do not present time result for Layer 3 because during the on-line mode, it runs as a different working thread on a different computation core, and it always requires less computation time than all of the other layers singularly considered. At the same time, as previously clarified, we do not present results for Layer 5, which was directly designed for the workstation.

As can be noticed from the table, each frame requires about 11 milliseconds to be processed when we run only the first layer of the application on the more powerful workstation. In all other cases, *i.e.*, when the application is running more layers or it is running on the embedded system, the computation is slower. A more accurate analysis shows that the most expensive layer is, in fact, Layer 1, where the background subtraction algorithm has to manipulate the input video frames, so dealing with a quite large amount of information. Layer 2 and 4 are much less expensive, as information is already filtered and the amount of data to manipulate is reduced. The embedded system runs at about 50% of the workstation speed, and even with all layers in place, it is theoretically able to reach about 21 frames per second. In practice, when we take into consideration all overheads (camera included, *i.e.*, the frame capturing time, *etc*.) the frame rate deteriorates a little bit, down to 17–18 fps. Anyway, in our context, experiments show that the final system counting accuracy does not deteriorate when at least 15 fps are manipulated by the system.

### 4.2. System Accuracy Evaluation

In order to demonstrate the accuracy of our system in optimal operational conditions, we present in this section some results obtained in two test scenarios. In this context “optimal operational conditions” means that the system has been evaluated on an “average” cloudy day (with standard illumination) and no traffic flow alterations. The analyzed scenarios include the roundabout (our running example) and the cross-road depicted in Figure 10.

In both cases, we ran the counting process for 1 h (54,000 frames, *i.e.*, about 15 frames per second). To assess its accuracy, we also manually counted the vehicles. While a stable flow model can be obtained in less than ten minutes, we decided to force the learning mode to run for a longer period (about 90 min) in order to obtain highly accurate models.

Table 2 and Table 3 show results for the roundabout and the cross-road scenario, respectively.

For each lane of the flow model (“Lane” column, labels are depicted in Figure 7 and Figure 10b), each table reports the number of vehicles counted by a human operator (“MC”, manual count), the number of vehicles counted by our system (“SC”, system count), the precision of our methodology (“accuracy”) and a label (the last column) stating whether a lane ends with a source (S) or a sink (SK) of the resulting flow network.

As for the accuracy, since labeling all individual frames was too complex in our case, we could not rely on standard evaluation metrics based on the number of true/false positives and negatives. Thus, given the manual count (MC) and the algorithm results (SC), we defined the accuracy for a lane *i* as:

(1) $$\begin{array}{ccc} {\mathrm{Accuracy}}_{i} & = & 1-\frac{|MC_{i}-SC_{i}|}{MC_{i}} \end{array}$$

The tables also present the overall sum of manually- and automatically-counted vehicles and the corresponding accuracy (“Total” row). We finally provide the actual number of outgoing vehicles (from the entire grid) as the sum of counted vehicles on sinks (“∑ Sinks” row) and, similarly, the number of (total) incoming vehicles (“∑ Sources” row).

Accuracy results for the two cases clearly show that the reliability of our system (under regular operational condition) is very high, both in terms of accuracy per lane and of overall vehicles detected.

### 4.3. Dealing with Non-Optimal Operational Conditions

**Figure 12:**
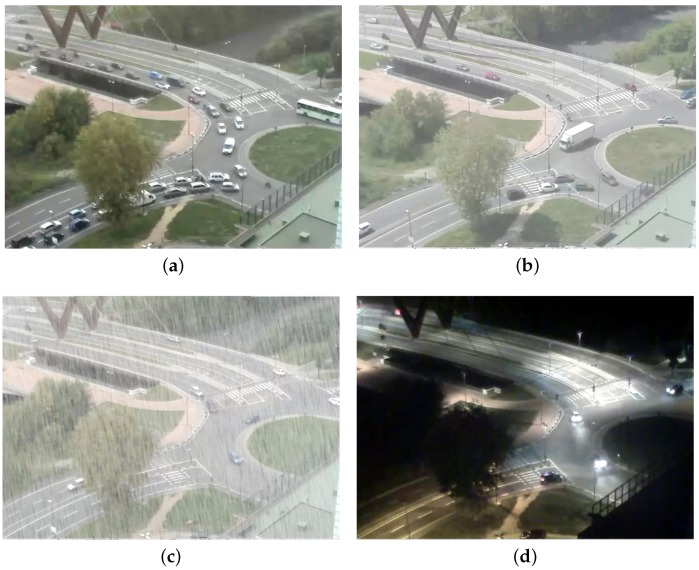
The roundabout running example in (**a**) cloudy, (**b**) sunny and (**c**) rainy/foggy conditions, as well as (**d**) during a night with standard weather conditions.

Varying operational conditions clearly affect the results of our system. In order to understand to what extent such variations influence the accuracy, we compared the results obtained in the two test scenarios described in Section 4.2 on three different weather conditions (cloudy, sunny, rainy/foggy) and during the night (Figure 12). In all of those cases, we analyze the scenes for 1 h.

The results of Section 4.2, obtained with cloudy conditions, clearly assure better accuracy and are, thus, used as a baseline. The flow models used for all conditions in the two scenarios were the ones obtained in Section 4.2. For the sake of brevity, we do not report detailed results for all lanes, but we simply compare their average accuracies. As can be seen in Table 4, the performances decrease, reaching, in both scenarios, the minimum value at night. As the minimum precision is still related to a considerable accuracy, we deem our system to be reliable in different operational modes.

### 4.4. System Sensitivity to Parameters

In Section 3, we introduced several parameters to describe the main features of our algorithm. In this section, we evaluate the sensitivity of our approach to these parameters. This is relevant information since, if the sensitivity is high, the fine tuning required to adapt the algorithm to different scenarios can become quite impractical. To this end, we conducted several experiments to analyze the influence of the different parameters on the system accuracy. These experiments were organized as follows. We (again) considered the roundabout as the main working scenario, then we fixed all parameters, but one, which assumed increasing values in a wide range. The sensitivity of the system to the main parameters can be described as follows:

- As illustrated in Section 3.1, background subtraction relies on *N*, the number of frames adopted to create the background model. We recall that higher values of *N* increase the adaptation rate of the algorithm, thus making it less responsive to abrupt changes. Results show that the higher the value, the better the accuracy. However, we found values around 200 to be adequate in all of our test cases.
- The threshold used to prune non-vehicle objects should be tuned for each camera. For instance, in the roundabout scenario, we found the optimal threshold to be 50 pixels.
- Flow model learning relies on two main parameters: the cell size and the threshold $t_{\alpha }$ used to create new cell directions. As for $t_{\alpha }$, we found similar accuracies for $t_{\alpha }\in \left[10,30\right]$ and optimal ones for $t_{\alpha }=30$; outside of this range, we experienced a sensible accuracy loss. As for the cell size, almost identical accuracies were obtained for sizes in the range [4,10] pixels, with a large advantage in terms of learning time (which for Size 4 is almost twice the time required at Size 10); larger cell sizes further improve the learning time in spite of a considerable drop of the accuracy. Summarizing, we fixed the cell size to eight pixels.

Counting and statistical layers do not have parameters significantly impacting either the accuracy or the execution time. To conclude, these experiments show that our system can be adapted to different scenarios with a minimal effort in the setup phase (*i.e.*, the setting of a single threshold for pruning non-vehicle objects).

### 4.5. Overall On-The-Road Results

**Figure 13:**
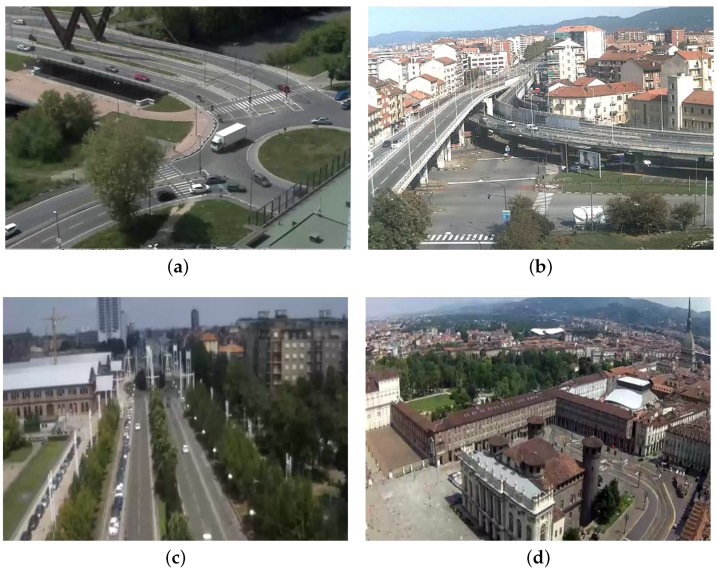
Counting scenarios: (**a**) Grosseto; (**b**) Mortara; (**c**) Castel Fidardo; and (**d**) Piazza Castello.

Concluding, to further show the overall robustness of the deployed system, we present counting results for the four different scenarios reported in Figure 13, namely “Grosseto” (the roundabout, Figure 13a), “Mortara” (the cross-road, Figure 13b), “Castel Fidardo” (a straight street, Figure 13c) and “Piazza Castello” (a large square, Figure 13d). Table 5 reports the number of lanes and the counting accuracy for these scenarios. Data have been collected during an entire day, *i.e.*, 24 h. They show that the average accuracies of our system are high for all cases, except Piazza Castello. This test case is interesting, since it allows one to stress the limits of our system for the following reasons. First, the camera is placed at a very high altitude, and it captures a twisted prospective of the square, such that all vehicles are extremely small and distorted. Second, a large portion of the image is occluded by buildings, which hampers the creation of an accurate flow model. Finally, the bottom-left region of the image frames a pedestrian zone, which is a large source of noise for the segmentation algorithm, since pedestrians have a size comparable to, but not larger than, moving vehicles.

## 5. Conclusions

In this paper, we have presented the design and the implementation of a real-time vision-based traffic flow monitoring system. The system is particularly suitable for low-cost, low-computational power embedded applications. The application is auto-adaptive, *i.e.*, it initially runs a training phase in which it learns the traffic model without requiring any human intervention, and it is capable of autonomously updating the model when variations of the traffic scheme have been detected. The software is easy to set up, since adjusting its parameters to different scenarios requires minimal effort. The overall system is based on a pipelined architecture where each computational layer (i) is kept as simple as possible in order to reduce its computational burden and (ii) is aimed at summarizing its input data in order to reduce noise and enable higher level processing in the following layer. Its final output is a coherent flow network model, which allows one to both obtain punctual evaluations and to compute a wide variety of traffic statistics. Experiments, run on real in-the-field scenarios for over two years and in different operating conditions, show that the approach is robust, precise and reliable.

## Figures and Tables

**Table 1:** Workstation *vs.* embedded system comparison: CPU time required by the computation performed by the different layers.

Operation Layer	Workstation (ms)	Embedded (ms)
Layer 1	11.45	20.44
Layer (1+2)	16.74	39.15
Layer (1+2+4)	19.33	46.79

**Table 2:** Accuracy for the roundabout scenario with optimal operational conditions. MC, manual count; SC, system count; S, source; SK, sink.

Lane	MC	SC	Accuracy (%)	S, SK
0	239	232	97.07	S
1	173	178	96.93	SK
2	51	57	88.24	SK
3	62	68	90.32	-
4	93	85	91.40	S
5	20	17	85.00	S
6	1354	1352	99.85	S
7	239	212	88.70	-
8	1482	1479	99.77	-
9	128	112	87.23	-
10	1482	1438	97.00	SK
11	173	169	97.68	-
**Total**	5496	5399	98.23	
**Sink**	1706	1673	98.06	
**Source**	1706	1686	98.83	
**Sink-Source**		−13		

**Table 3:** Accuracy for the cross-road scenario with optimal operational conditions.

Lane	MC	SC	Accuracy (%)	S, SK
0	622	593	95.34	S-SK
1	704	672	95.45	S-SK
2	353	344	97.45	S-SK
3	489	495	98.77	S
4	216	235	91.20	SK
5	970	964	99.38	S-SK
6	861	842	97.79	S-SK
7	93	98	94.62	SK
8	360	356	98.89	S
9	746	784	94.91	SK
10	746	725	97.18	S
11	144	162	87.50	SK
12	228	218	95.61	S
13	273	256	93.77	SK
14	216	196	90.74	SK
15	135	127	94.07	SK
**Total**	7156	7067	98.76	
**Sink**	5333	5273	98.87	
**Source**	5333	5209	97.67	
**Sink-Source**		64		

**Table 4:** System average accuracy with non-optimal operational conditions.

Operational Conditions	Roundabout (%)	Cross-Road (%)
Cloudy	98.23	98.76
Sunny	90.05	91.71
Rainy/Foggy	84.91	85.04
Night	83.08	84.86

**Table 5:** Counting results for different scenarios.

Scenario	# of Lanes	Accuracy (%)
Grosseto	12	96.83
Mortara	16	96.80
Castel Fidardo	2	97.11
Piazza Castello	5	53.51
